# Artificial Intelligence for Colonoscopy: Past, Present, and Future

**DOI:** 10.1109/JBHI.2022.3160098

**Published:** 2022-08-11

**Authors:** Wallapak Tavanapong, JungHwan Oh, Michael A. Riegler, Mohammed Khaleel, Bhuvan Mittal, Piet C. de Groen

**Affiliations:** Iowa State University, Ames, IA 50011-2140 USA; University of North Texas, Denton, TX 76203 USA; SimulaMet, Oslo, Norway and UiT The Arctic University of Norway, 9019 Tromsø, Norway; Iowa State University, Ames, IA 50011-2140 USA; University of North Texas, Denton, TX 76203 USA; University of Minnesota, Minneapolis, MN 55455 USA

**Keywords:** Artificial intelligence, medical image analysis, real-time systems, machine learning, colonos copy

## Abstract

During the past decades, many automated image analysis methods have been developed for colonoscopy. Real-time implementation of the most promising methods during colonoscopy has been tested in clinical trials, including several recent multi-center studies. All trials have shown results that may contribute to prevention of colorectal cancer. We summarize the past and present development of colonoscopy video analysis methods, focusing on two categories of artificial intelligence (AI) technologies used in clinical trials. These are (1) analysis and feedback for improving colonoscopy quality and (2) detection of abnormalities. Our survey includes methods that use traditional machine learning algorithms on carefully designed hand-crafted features as well as recent deep-learning methods. Lastly, we present the gap between current state-of-the-art technology and desirable clinical features and conclude with future directions of endoscopic AI technology development that will bridge the current gap.

## I. Introduction

Over the past two decades, automated analysis of endoscopic images recorded during colonoscopy has become a research area of great interest. Colonoscopy is the gold-standard for prevention of colorectal cancer (CRC), because during colonoscopy endoscopists can examine the entire colon and remove all premalignant lesions. Therefore, timely enrollment in a colonoscopy-based screening program in principle should prevent most CRC. Yet, despite stool-based and colonoscopy-based screening programs in many countries, CRC still causes significant morbidity and mortality [1]. In 2020, there were 935,173 deaths worldwide [1] and around 53,200 deaths in the U.S. [2].

**Fig. 1.**
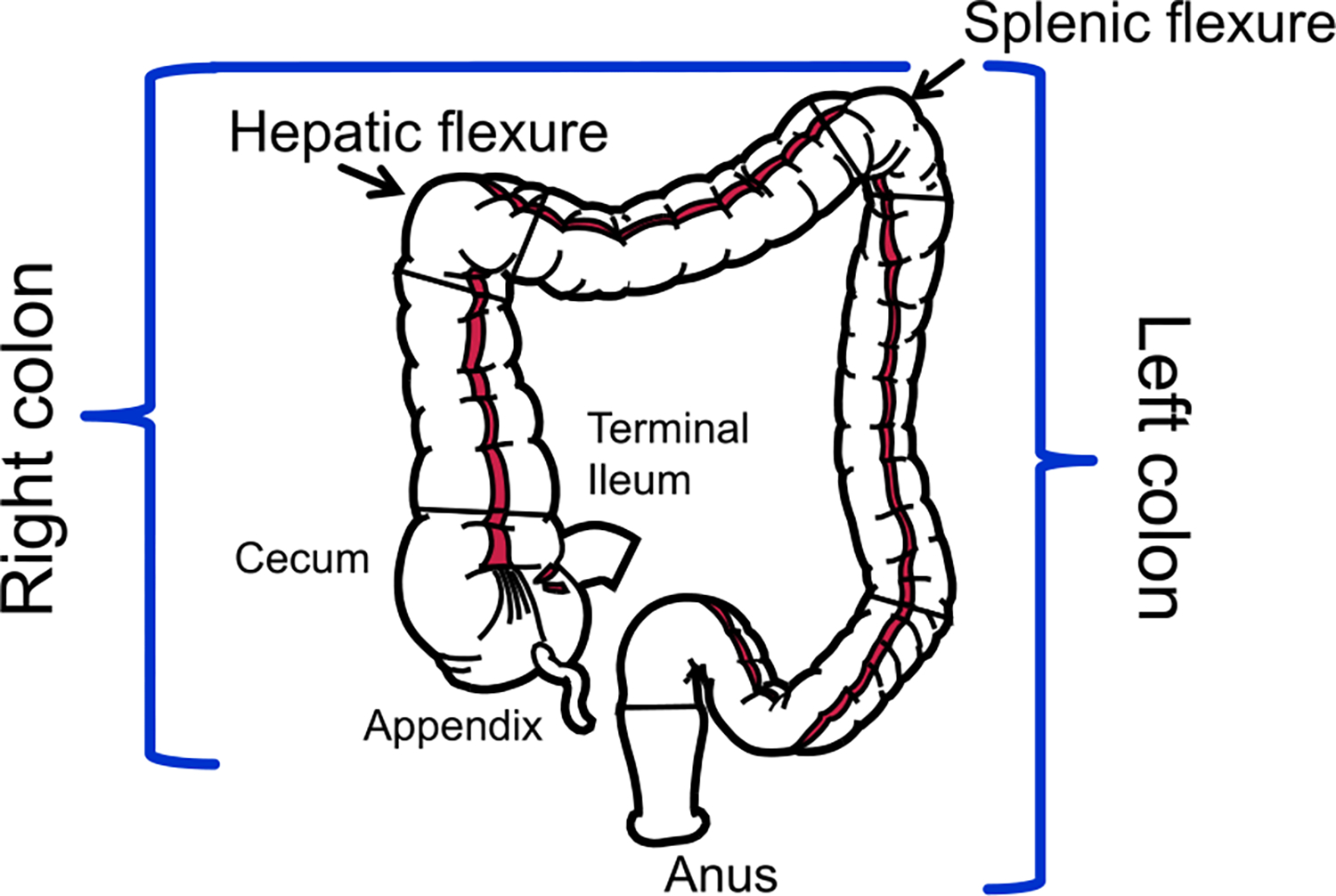
Diagram showing the colon anatomy.

The colon is about five feet (150 cm) long and nested inside the human abdomen. Fig. 1 shows the anatomy of the colon. In the first phase of colonoscopy the endoscopist advances a flexible endoscope with a single wide-angle camera lens at the tip from the anus upstream with the intent to reach the cecum. The second phase starts at the point of maximum intubation; from this point the endoscope is gradually withdrawn. Careful examination behind colon folds and angulations is performed during the withdrawal phase by flexing the tip and torquing the shaft of the instrument to maximize mucosal coverage and avoid missing any abnormality located outside the longitudinal or axial view with the tip of the instrument in the neutral, straight position. At the same time premalignant lesions are removed. Both inspection and removal of lesions can vary from easy to difficult; successful completion of both, especially within a limited time, requires an advanced skill set, which explains why colonoscopy is an operator-dependent procedure.

In the early years of colonoscopy image analysis, image processing was typically used to extract carefully designed features as input to traditional machine learning methods for decision making. The last decade has seen a significant growth in supervised deep-learning (DL) methods for colonoscopy with automated feature learning from raw training images for prediction. Two surveys focusing on development of analysis methods [3], [4] were written by computing researchers. Readers interested in analysis methods for colonoscopy including pre- and post-procedure analysis (e.g., content-based video retrieval, efficient storage, efficient video interaction and browsing) as well as analysis for other types of minimum invasive endoscopy surgeries are referred to [3]. Readers with interest in deep-learning methods for polyp image detection, polyp region localization and segmentation prior to 2020 are referred to [4]. The latter survey also includes information about publicly available polyp datasets and performance metrics.

**Fig. 2.**
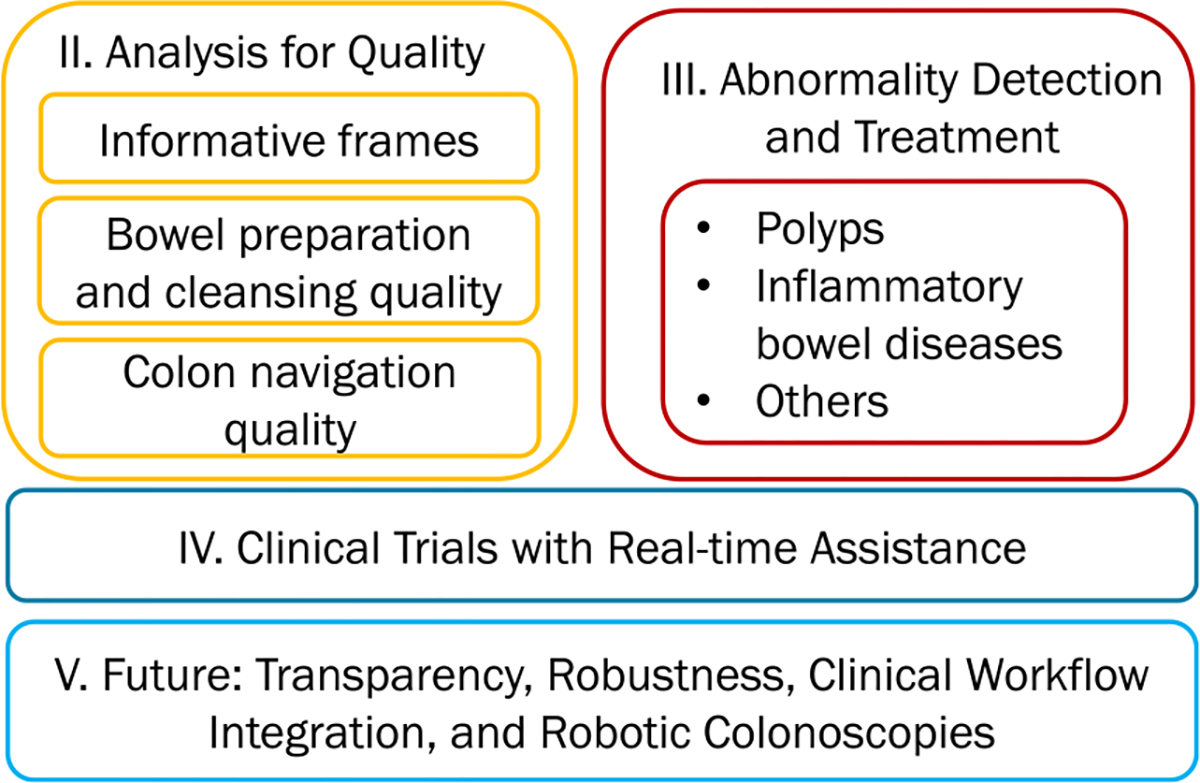
Overview of the topics (in Sections II–V) summarized in this survey.

Unlike [3], this survey focuses on methods aimed for real-time assistance during live colonoscopy procedures. It does not cover analysis for wireless capsule endoscopy [5] or other types of endoscopy procedures [3]. Unlike [4], we summarize methods beyond polyp detection, localization, and segmentation. Polyps must first appear in the field of view of the camera before any image analysis methods can find them. This requires a good bowel preparation by the patient and most importantly good quality inspection skills by the endoscopist after reaching the cecum. That is to (1) clean remaining fecal debris, (2) see adequate amount of frames in focus (non-blurry frames), (3) look everywhere behind folds and difficult to reach areas, and (4) perform high quality, complete polypectomy [6]. Fig. 2 outlines the topics discussed in this survey.

The future of AI-assisted colonoscopy was forecast by leading domain experts in their surveys [7]–[11]. They agree that AI systems for endoscopy are forthcoming, and anticipate that AI-assisted polyp detection systems will become widely available clinically in the next five years [8]. GI Genius owned by Cosmo Pharmaceuticals and commercialized by Medtronic received FDA approval for use in the U.S. in 2021 and this system is already in place in some hospitals. ODIN Vision’s CADDIE is in an on-going clinical trial in the U.K. [12]. However, the domain experts also expressed concerns about deployment in clinical practice. We categorize these concerns into **robustness**, **transparency**, and **cost-effective integration** of AI systems into clinical workflow.

We use the term “robustness” broadly to cover a number of issues. The training datasets reported in the literature are much smaller than the amount of data generated during routine colonoscopy screening and may not represent the real world. Training images tend to represent optimal conditions, e.g., a picture with a clean colon in perfect focus. *How well does the model pre-trained on small datasets under optimal conditions generalize to real-world data under sub-optimal conditions e.g., polyps partially occluded with feces?*

We use the term “transparency” to include adequate disclosure about ground truth training data such as the number of training images, the diversity of the training data, inherent biases in the training data, and explainability of deep models in making predictions. Our contributions are as follows.

- We summarize existing research aimed for real-time assistance during colonoscopy in three subcategories: analysis of the quality of the colon inspection, analysis for abnormalities and treatment, and the clinical trials using real-time AI-assisted technology. The summary of the quality of the colon inspection methods and the feedback used in clinical trials were not included in the existing surveys.
- As deep learning models are prevalent in present and future AI systems for colonoscopy, it is important to focus on improving robustness and transparency of deep-learning models for colonoscopy in clinical use. This topic has received the least research attention and was not included in detail in the existing surveys. We summarize existing methods that were applied to colonoscopy.
- We discuss future research directions including robustness and transparency, integration with clinical workflow, and robotic colonoscopies.

Many methods were proposed and evaluated over the years. Because there are few publicly available annotated datasets, many researchers used their private datasets for performance evaluations. The available public datasets [13]–[18] are mostly for polyp detection and segmentation. They are relatively small and have images taken under an optimal condition. They do not yet represent a large variety of colonoscopy images in clinical use. Due to these limitations, we do not compare existing methods directly, but present them in a chronological order. Except the topic of colon navigation techniques via 3D reconstruction, we also omit performance reports based on evaluations using small private test datasets (i.e., fewer than 3,000 images or fewer than 10 full length colonoscopy videos).

## II. Analysis for Objective Quality Measurements

Objective measurements of quality of colonoscopy are important to reduce subjective biases and differences among endoscopists [19]. We focus on three key measures of quality of colonoscopy [20]: the amount of blurry (non-informative) images during the withdrawal phase, the quality of bowel preparation by patients prior to colonoscopy and the effort to remove remaining debris by the endoscopist, and the quality of the endoscope navigation inside the colon. The latter remains very challenging to solve, but has recently gained more interest due to its significance to the clinical outcome.

### A. Informative Frame Analysis

An informative frame in a colonoscopy video can be broadly defined as a frame in focus and useful for analysis of the colon mucosa [21]. If most frames during the withdrawal phase of the procedure are non-informative or blurry, then a significant part of the mucosa may not have undergone adequate inspection. Furthermore, distinguishing non-informative frames from informative ones early can improve accuracy of analysis of colonoscopy video frames for other purposes such as detection of abnormalities. Several features can distinguish non-informative frames from informative ones: corner and edge features matched with the previous frame, the percentage of edge pixels, and the mean and standard deviation of intensity in HSV (hue-saturation-value) color space were investigated in [22]. A Random Forest classifier was used for classification. An enhanced edge detection-based method was proposed in [23], [24]. Non-Informative frames usually do not contain many edges. However, very bright regions due to specular reflections can produce false edges. Therefore, the proposed method includes bright region segmentation to identify and remove false edges.

A Convolutional Neural Network (CNN) model was used for the first time for this problem in [25]. Inadequate or improper bowel preparation is characterized by remaining debris and cleansing agent which are causes of non-informative frames. SimpleNet (CNN implemented from scratch by the authors), AlexNet [26], GoogLeNet [27] and ResNet [28] were compared in terms of accuracy and speed using a dataset of about 12,000 frames. The experimental results showed that the CNN methods were fast at detecting non-informative frames with accuracies of 70 to 95%.

Hand-crafted and deep learning features from a pre-trained Inception-v3 model were combined in [29] to classify non-informative images. Although the required computation time was high, experiments based on around 17,000 frames showed an average Area-Under-the-Curve of 93.9% and an average F1 score of 77.5%. Resnet18 with Long-Short-Term-Memory (LSTM) or Gated-Recurrent-Unit (GRU) was proposed to learn from the temporal sequence of frames to predict the informativeness [30]. Gradient-weighted Class Activation Map (Grad-CAM) [31] interpretation was used to localize the informativeness within a frame. The Resnet18 extracted features were input to three separate classifiers, namely, the fully connected network, LSTM, and GRU.

### B. Bowel Preparation and Cleansing

Bowel preparation (cleansing) is a key precondition for a successful colonoscopy. The degree of bowel cleansing affects successful disease detection. Therefore, an accurate assessment of bowel preparation quality is important. The Boston Bowel Preparation Scale (BBPS) [32] is a widely used bowel preparation quality assessment score. BBPS measures the individual cleanliness of three colon segments (ascending colon, transverse colon and descending colon) with a score ranging from 0 (dirtiest) to 3 (cleanest); the addition of the segmental scores provides the overall BBPS score.

Informative frames were classified by Support Vector Machine (SVM) into frames with and without remaining debris in [33]. A CNN with two DenseNet layers which have a feature reuse mechanism embedded before the softmax classifier was proposed to estimate BBPS scores [34]. This method achieved an accuracy of 90% based on the public Nerthus dataset [16]. EndoAngel based on a CNN architecture outputs bowel preparation scores every 30 seconds during the withdrawal phase of colonoscopy [35]; an accuracy of 89% was achieved over 20 colonoscopy videos.

### C. Analysis of Navigation Quality

Circumferential or 360 degrees inspection of the colon mucosa throughout the withdrawal phase of colonoscopy leads to high quality of colonoscopy and greatly reduced mortality from CRC [6]. A few objective measurements have been proposed and analysis methods to derive these metrics were introduced.

#### 1) Inspection Coverage of Colon Mucosa:

**Fig. 3.**
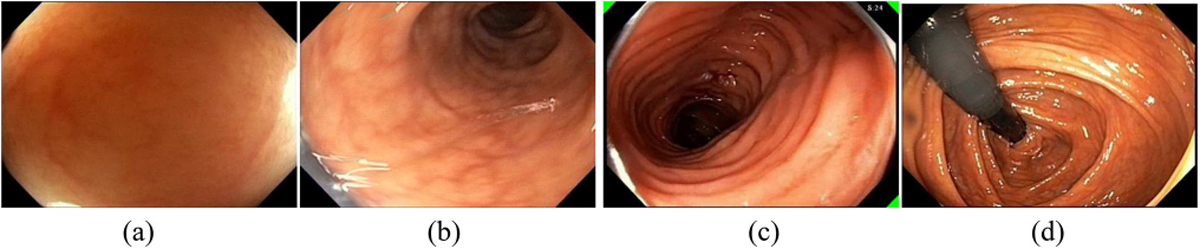
Examples (a) wall view; (b) lumen view; (c) spiral score and feedback; (d) retroflexion for viewing a difficult-to-reach area.

Liu *et al.* [36] proposed the first objective metric called “Quadrant Coverage Histogram (QCH)” based on the domain knowledge that both distant and close up inspection should be performed during the withdrawal phase of colonoscopy. To compute QCH, an SVM classifier separates informative frames into two classes: “wall view” and “lumen view”. Wall views are informative frames without the lumen, which represents close up inspection of the colon wall (Fig. 3(a)). Lumen views are informative frames with the colon lumen seen in the distance. Given a lumen view, the quadrant of the colon the endoscopist is focusing on is estimated to be the opposite quadrant where the lumen locates. For instance, in Fig. 3(b), the lumen is in the top right quadrant and the inspected quadrant is the lower left quadrant. QCH score is the average number of quadrants seen in a given duration (time window). A QCH of one indicates that only one side of the colon is inspected by the endoscopist.

Later on, “spiral score” was proposed [37] where a “spiral” is defined as a completion of inspection of four different quadrants of the colon considering only the lumen views. The spiral score is a count of the number of “spirals” performed thus far. The more “spirals,” the more likely a high-quality inspection of the colon. Fig. 3(c) shows the spiral score as the white text on the top right corner and three little green triangles indicating the quadrants that had been inspected. Hong *et al.* improved the method for calculating spiral scores [38] based on detection of colon fold edges and the center of the innermost haustral fold. Feedback showing the spiral scores was used in a single center clinical trial on ten GI-trainees [39] over 159 colonoscopy procedures. The study found the spiral score feedback resulted in statistically significant improvement in quality of the colon examination. The spiral score was one of the key factors of the first single automated score of quality of colonoscopy. The automated score was developed using a mixed stepwise logistic regression model and validated on 200 full colonoscopy procedures [40].

The spiral score is a coarse estimation of how well the endoscopist looks everywhere during colonoscopy. Several attempts were made to obtain a more detailed estimate via 3D reconstruction of a virtual colon from a sequence of colonoscopy images. None of techniques have reported real-time performance in live colonoscopy procedures. The research challenge is the lack of detailed ground truth of camera depths and motion parameters during colonoscopy.

Zhou *et al.* [41] proposed a method to generate a small 3D colon segment by using optical flow analysis to align neighboring 3D circles (approximating colon folds) and determine the distance among them. The limitations of this method are as follows. Some colon segments (e.g., transverse colon) are not circular. Partial occlusion of colon folds is typical in practice. Lastly, colon fold thickness is not modeled, which is important as polyps may be hidden behind folds. Two methods for 3D reconstruction given a single image [42] and sequential images [43] were proposed. These techniques do not have the aforementioned limitations. For reconstruction from a single image, the method [42] first estimates colon fold contours and places the detected folds in 3D space via reverse projection and depth estimation from non-specular pixels. Next surface of the colon folds and surface between folds are generated to complete the reconstruction of a 3D virtual colon segment from a single image. The percentage of the colon mucosa area not seen in the field-of-view of the camera and a 3D map of the unseen areas are estimated from a reconstructed 3D colon via a simulation of a simple fly through inside the virtual colon from the first colon fold to the last fold without lateral tip deflection [44]. These metrics are of interest to objectively estimate how well the endoscopist inspects the colon and which areas have not been inspected. The method in [43] tracks detected colon fold edges across a sequence of images and reconstructs a corresponding 3D colon segment and camera motion parameters.

Mahood and Dur proposed a deep-learning method that reconstructs 3D surface of a colon from a single image [45]. The proposed network takes an over-segmented input image and outputs the predicted depth map. The network architecture consists of three major components. One component consists of five convolutional layers followed by four fully connected layers. Another component is a fully connected layer that takes neighborhood pairwise superpixel similarities from the over-segmented input image. The output of both components are input to the third component—the conditional random field layer. In [46], the authors improved upon their previous method [45] using a fully convolutional network to generate convolutional feature maps and nearest neighborhood upsampling to generate superpixel feature vectors.

Colonoscopy Coverage Deficiency via Depth (C2D2) was proposed to predict the colonoscopy coverage [47]. Coverage score per colon segment was defined as a fraction of the colon mucosa in the field of view of the camera to all visible mucosa area in a colon segment. The colon segment model is simplified by excluding fold thickness from the model. Hence, the mucosa area behind folds is not taken into account in the calculation of the coverage score. C2D2 uses ResNet-18 to estimate a depth image directly from an RGB input image and estimates camera intrinsics as well as the camera pose (translation vector and rotation matrix) between this frame and its preceding frame. To predict the coverage score for a segment, two additional neural networks are used. The first network is a 2D CNN (ResNet-50) with the global spatial average pooling (GAP) layer before the fully connected (FC) layer. The second network takes a sequence of frame feature vectors output by GAP of the first network and predicts the coverage score for a sequence of 300 frames. This network has a 1D CNN followed by GAP (in the temporal domain) before the FC layer. The total time for all stages was less than 17.07 ms per frame. The Google-Synthetic dataset used is available upon request; it has 187,369 (RGB, depth) image pairs with a train-test split of 134,025 and 53,344, respectively [47]. For qualitative evaluation, two domain experts were asked whether they agreed with the reconstruction results on 301 real colonoscopy sequences. Each sequence was rated by one domain expert. The experts agreed with the results in 93% of the sequences.

Armin *et al.* [48] proposed a CNN that predicts the colon center line (a set of points in the middle of the colon lumen) and camera direction from a sequence of colonoscopy frames. The network is based on VGG16, but takes a pair of consecutive frames as input. By modeling a colon segment as a cylinder, a colonoscopy frame is projected onto the cylinder and unrolled into a radial strip called a “band image”. Band images of consecutive frames are then stitched together based on average motion flows to form a “visibility map”. Ma *et al.* [49] proposed RNNSLAM integrating a localization and mapping method and depth and pose estimation neural networks to reconstruct 3D colon segments. Blau *et al.* [50] proposed an unsupervised learning technique for estimating examination coverage on colon segments modeled as bent cylinders. The work by Zhang *et al.* [51] and Mathew *et al.* [52] utilizes pre-procedure CT scans for reconstruction of 3D colon segments. Abrahams *et al.* [53] proposed to predict blindspots at acute bends in the colon assuming a known colon centerline, the camera’s pose relative to the model, and a torus colon model with fixed-diameter circular cross-sections and straight or bent centerline. Lastly, Ma *et al.* [54] made available a Colon 10K dataset for evaluation of methods for finding the region in the colon in the current colonoscopy given an image taken from the same patient in a previous colonoscopy.

Several challenging research problems remain, for instance, 1) modeling deep haustral folds where polyps may be hidden, 2) handling low-texture and intensity variations, and presence of instruments, debris, and water, 3) modeling non-circular colon segments and geometric distortion of the colon, and 4) quantitative evaluation of the reconstruction of the colon from a full-length colonoscopy procedure since there is no quantitative ground truth of the true structure of the colon during colonoscopy.

#### 2) Retroflexion Detection:

Retroflexion is an endoscope maneuver where the tip of a flexible endoscope equipped with a wide angle lens is deflected more than 90 degrees from the axial direction of the shaft of the endoscope. Retroflexion allows examination of the colon in difficult to reach areas such as the hepatic flexture and peri-anal mucosa in the rectum. Fig. 3(d) shows retroflexion in the right colon. Rectal retroflexion was suggested as an essential part of the colonoscopy examination [55]. Studies reported that retroflexion improved the yield of polyps [55]–[57]. The meta analysis study [55] of six studies compared colonoscopy with right-sided retroflexion and without. The study concluded that retroflexion in the right colon improved the detection of adenomas in the right colon and recommended that it be strongly considered in the guidelines for standard of care for colonoscopy [55]. The challenge for detecting retroflexion automatically is the short duration of some retroflexions (about 1–2 seconds) and the dark appearance of the endoscope within a dark lumen. During retroflexion, the endoscope may be bent, appear in gray color or blurry due to rotation of the scope, or be partially occluded from view. The scope may also appear in a small portion of the screen blending with the black background at the edge of the endoscopic field of view. Wang *et al.* proposed pre-processing steps and hand-crafted features as input to SVM and Decision Tree classifiers to predict whether an image shows retroflexion or not [58]. Although promising, the required compute time did not allow real-time detection. Thus better methods are needed for real-time detection and quality estimation of retroflexion. For AI systems with a focus on quality of colonoscopy, an accurate estimation of amount or percent of all mucosa seen posts technical challenges, mainly due to variation in individual colon shape, the unpredictable nature of colonic contractions, and a lack of ground truth for training and verification of new AI methods. Estimates of effort of inspection, such as the spiral score, a coarse heat map of inspected mucosa or detection of retroflexion in the right colon and rectum, are starting to address the critical issue of mucosal coverage. However, more detailed methods combining new imaging with advanced mapping and AI-based interpretation systems that include AI-assisted polyp detection are needed to provide more detailed objective evidence of amount of colonic mucosa seen and number of polyps present.

## III. Abnormality and Treatment Detection

### A. Polyp Detection and Segmentation

Colon polyps are generally classified based on their appearance as pedunculated, sessile, or flat. Pedunculated polyps have short or long stalks. Sessile polyps grow on the surface of the colon without a stalk. Flat polyps grow along the surface of the colon. In general, sessile polyps, the head of pedunculated polyps, and flat polyps have an elliptical shape when small. Some polyps may transition into CRC. Complete removal of polyps during colonoscopy prevents the transition to CRC. Polyps vary in their appearance, shape, size, amount of protrusion, and location in the colon; to complicate matters, the same polyp may appear differently in different images due to amount of colon insufflation, degree of colon muscular contraction, angle of view, and distance from the camera. Objects between the lens such as remaining debris or instruments, may prevent polyp visualization.

Detection of colon polyps using computer assisted methods has been an active topic for research over the last two decades. During that time the focus has shifted from proof of concept work toward real-time deployment; e.g., how to achieve high detection rates while maintaining high precision in real-time [4], [59]–[61]. Early on research was focused on polyp features such as shape, color, and texture. Most methods consisted of feature engineering and used the handcrafted features for learning [62], [63]. That changed around 2016 when methods based on deep neural networks, in particular CNNs, were applied to polyp detection [64]–[67]. Performance comparison studies were reported in [4], [66], [68]. One of the most influential and first works was done by Wang *et al.* [59]. They presented algorithms and software modules for near real-time polyp detection. In addition to the algorithm a software system called Polyp-Alert was presented, which was the first complete system for automatic polyp detection. Since this report, many other studies have been completed. YOLO and similar methods [69] use deep-learning architecture for detection and localization of colon polyps. Different implementations of YOLO are mostly known and applied for their real-time capabilities. For example, Lee *et al.* [70] used YOLOv2 in their polyp detection and localization algorithm. Wan *et al.* used the latest YOLOv5 to [71] to perform polyp detection. Both articles show that YOLO-based methods have good sensitivity and near real-time performance.

Once accurate automated detection and localization of polyps was achieved, research efforts focused on pixel-wise classification or segmentation methods. Segmentation methods are intended to provide exact polyp boundaries and use every single pixel of a polyp for training. Therefore, smaller datasets can be used for training. Jha *et al.* [72] proposed a new architecture, ResUNet++. They also proposed a DoubleUNet architecture for solving the segmentation task. For a polyp segmentation task, performance metrics include Dice Coefficient, Jaccard Coefficient, precision, recall, and overall accuracy [60]. DoubleUNet is a combination of two stacked U-Nets [73] and variations of this architecture are commonly used for polyp segmentation [74]–[78]. Others used fully convolutional dilation networks to perform the analysis [79], [80]. Ali *et al.* [67] evaluated segmentation approaches against their robustness for artifacts that are part of clinical endoscopy videos and images [81].

A boundary-aware network (BA-Net) for segmentation was proposed by Wang *et al.* [82]. The architecture is based on an encoder-decoder network which captures high-level context and at the same time preserves spatial information. In [83], [84] also boundaries are taken into account to improve U-Net-based architectures. The main goal of boundary-based approaches is to take into account the information of the boundary of polyps compared to the polyp itself. Polyp segmentation using SegNet, a deep learning based segmentation model, can process around 25 frames per second [85], [86], which is seen as the border for real-time feedback during colonoscopy. Bernel *et al.* [66] compared the performance of eight different methods for polyp localization and segmentation and provided an analysis of various detection methods. Their best overall performance was a precision of 85.6,% a recall of 76.8%, and an F1 score of 81%. Their work was based on a dataset of 38 videos (20 training, 18 testing) with many near-duplicate frames. Puyal *et al.* [87] proposed a hybrid 2D/3D CNN to take advantage of both spatial and temporal information.

There are many more recent approaches and most also rely on the well-known U-Net architecture as a basis with different modifications or in different variations [88]–[91]. Interest in image segmentation of polyps remains very high and new work is appearing almost daily [92]–[100]. Generalizability of the models for polyp segmentation has become an important factor to consider. The Polyp Segmentation challenge 2021 (EndoCV 21) provided a new dataset that consisted of polyps from different centers to specifically address generalizability. Two new architectures by Thambawita *et al.* [101] performed best in the challenge. One was a triple U-Net (TriUNet) consisting of three U-Nets combined. The second one is called DivergentNets and is a combination of five different segmentation networks where each of the networks learns a different view on the data. The DivergentNet method achieved an Intersection-Over-Union or Jaccard Index of 97.6%, an F1 score of 98.6%, a recall of 98.6%, and a precision of 98.6%.

Most of the polyp segmentation datasets are rather small in terms of the number of different polyps or the number of total frames or videos. In the EndoCV 21 challenge, the PolypGen dataset was used [102]. It contains data from six different clinics and more than 300 patients. In total 3,446 annotated polyp labels with precise segmentation masks of the polyps are included. All have been verified by six senior gastroenterologists. The best reported Dice coefficient on this dataset is around 82%, implying that there is still room for improvement [102]. Kvasir-SEG is another diverse, large dataset with segmentation for 1,000 different polyps [18]. Works that performed segmentation on the Kvasir-SEG dataset report a mean Dice coefficient between 0.787 [18] and 0.918 [103].

Considering the vast amount of research on polyp segmentation, it is challenging to keep track of the open problems and what the real improvements are. Based on insights from the articles referenced in this survey, we identified the following open challenges. (1) Generalizability of segmentation methods needs to be improved. (2) Current metrics are not representing performance requirements for clinical practice. (3) Segmentation datasets are still small and they do not often represent different centers/cameras. The datasets are often imbalanced or not diverse enough, in addition to the lack of the clinical outcome for many cases. Even if performance metrics indicate great performance of most of the proposed methods, it is not clear how this performance translates into clinical practice and how it relates to imbalanced data, which is an important gap that needs to be addressed by the community.

### B. Detection of Inflammatory Bowel Diseases

Ulcerative colitis (UC) is a chronic inflammatory disease of the large intestine which may extend upstream from rectum to cecum. It is characterized by periods of relapses and remissions affecting more than 750,000 in North America [104]. The therapeutic goals of UC are to first induce and then maintain disease remission. Endoscopic disease severity may better predict future outcomes of UC than symptoms. The challenges to evaluate the severity of UC objectively are non-uniform nature of symptoms associated with UC, and large variations in their patterns [105]. To assist UC diagnosis, Nosato *et al.* proposed a method [105] that uses geometrical features such as the textures of the colonic mucosa and their appearance in the colonoscopy images. The features are expressed by Higher-order Local Auto-Correlation (HLAC) [106] and Multivariate Data Analysis for classification of UC severity levels. In addition, a color conversion technique is used to enhance the ability to efficiently observe the colon conditions. Nosato *et al.* also proposed a method [107] to retrieve multi-scale objects related to UC from colonoscopy images based on HLAC. This method generates integral HLAC feature tables that are calculated using the HLAC extraction method [108].

To extract distinct textures for UC severity classification, a hybrid approach [109] uses a feature based on the accumulation of pixel value differences in combination with an existing feature such as Local Binary Pattern. A K-nearest-neighbor classifier was used to classify images into five categories: Severe, Moderate, Mild, Scar, and Normal.

Alammari *et al.* proposed a CNN-based method to objectively classify UC severity levels [110]. The first step classifies a frame into one of the ‘severe’, ‘moderate’, ‘mild,’ and ‘normal’ classes, and calculates the severity score automatically for a given video based on these classification results. Around 50,000 frames and 15,000 frames were used to train and test their CNNs, respectively. The frame-level test accuracy of 45% was reported to classify four classes.

Tejaswini *et al.* [111] proposed an improvement of [110] in two ways for better accuracy. First, essential preprocessing was added to discard out-of-focus frames, and frames containing large amounts of water or bubbles, excessive specular reflection areas, or very high uneven illumination. Second, each class of UC severity was subdivided, and more classes were generated to accommodate large variations in patterns. Each of three classes of UC such as ‘Mild’, ‘Moderate’, and ‘Severe’ are subdivided to ‘blood’ and ‘non-blood’ classes based on the amount of blood appearing in a frame. ‘Normal’ class is not divided to ‘blood’ and ‘non-blood’ classes since it is not ‘Normal’ if it includes any amount of blood. Thus there are a total of seven classes: ‘Normal’, ‘Mild-blood’, ‘Mild-non-blood’, ‘Moderate-blood’, ‘Moderate-non-blood’, ‘Severe-blood’, and ‘Severe-non-blood’. In the next step, each of these seven classes are subdivided to ‘flat’ and ‘non-flat’ classes based on the visual contents from different viewing directions. The proposed CNN has these 14 classes to classify into the four UC classes. Around 30,000 frames and 15,000 frames were used for training and testing, respectively. The frame-level test accuracy of 61% was reported for the four class classification, which is a 15% improvement over [110]. A method to classify UC severity by detecting the vascular (vein) patterns which are defined as the amount of blood vessels in a frame was proposed [112]. To detect these vascular patterns, image pre-processing methods and three CNNs were used for classification for four UC severity levels. Around 53,000 frames and 15,000 frames were used for training and testing, respectively. The frame level test accuracy of 80% was reported, which is a 19% improvement over their previous work [111].

A GoogLeNet based model was trained using 26,304 colonoscopy images from a cumulative total of 841 patients with UC [113]. The area under the receiver operating characteristic (AUROC) was used to evaluate CNN performance in classifying the normal mucosa and mucosal healing states (mild) using an independent test set of 3,981 images from 114 patients with UC. The study showed a high performance with AUROCs of 0.86 and 0.98 to identify normal and mild, respectively. However, this work did not consider the clinically important differences among mild, moderate and severe UC classes. To classify four different degrees of severity of the colonoscopy images with ulcerative colitis, a method using Efficient Attention Mechanism Network (EAM-Net) [114] and UC-DenseNet [115] was proposed [116]. Using 14,306 colonoscopy images, the accuracies were improved from 1% to 7% compared to the existing methods.

### C. Detection of Other Types of Abnormality

In [117], a SVM based method to classify normal and abnormal colonoscopy images was proposed. It uses the image-to-class (I2C) distance measure [118] for calculation of distances among the classes. Also, it uses an extension of LBP (Local Binary Pattern) called ‘discriminative feature learning’ to extract the input features for SVM, which is a combination of distance metric learning [119] and discriminative subspace learning [120].

To detect colon diseases, a combination of Cross-Wavelet Transform (XWT) [121] and MSVM (Multiclass Support Vector Machine) was proposed in [122]. XWT is an extension of a conventional wavelet transform, which outputs high dimensional features. Principal Component Analysis was used to reduce the feature dimensions for MSVM.

To distinguish abnormal images with lesions that need resection (adenoma and serrated adenoma), a method using features extracted from color, texture and morphology (3D shape) of the lesions was proposed [123]. The color-GLCM (Gray Level Co-occurrence Matrix), Invariant Local Binary Patterns [124], Invariant Gabor Texture Descriptors [125], and 3D configuration Shape-from-Motion [126] features were investigated.

Narrow Band Imaging (NBI) is a video endoscopic system that uses RGB rotary filters placed in front of a white light source to narrow the bandwidth of the spectral transmittance. It provides a limited penetration of light to the mucosal surface, and enhances the micro-vessels and their fine structure on the colorectal surface. The NBI International Colorectal Endoscopic (NICE) classification system divides NBI images into Types 1–3 based on three characteristics: (i) lesion color; (ii) microvascular architecture; and (iii) surface pattern. Type 1 includes hyperplastic lesions, Type 2 includes adenoma or mucosal/submucosal scanty invasive carcinoma, and Type 3 includes deep submucosal invasive carcinoma. Kuo *et al.* proposed a two-layered SVM classifier that separates NBI images into these three types [127]. It uses the features derived from the Bank of Binarized Statistical Image Features [128].

Shang *et al.* trained multiple 121-layer DenseNet models [115] with different combinations of five training datasets (NBI Colonoscopy, white-light Colonoscopy, Esophagogastroduodenoscopy, Skin Lesion, and ImageNet) [129]. The test dataset defines non-adenomatous polyp images as benign and adenomatous polyps and cancer images as malignant. A model using MobileNetV2 [130] and DenseNet-121 [115] was proposed [131] to detect abnormalities. A summary report about the main findings from videos of gastrointestinal (GI) tract examinations can be generated using Class Activation Maps [132].

For medical image classification, a combination of data augmentation, multi-epoch fusion, and adaptive threshold selection was proposed in [133]. Data augmentation methods were randomly selected from RandomContrast, RandomBrightness, RandomGamma, Blur, MotionBlur, InvertImg, Rotate, or RandomScale. For multi-epoch fusion, the weights of each layer in the last four epochs were averaged to generate the final model. In adaptive threshold selection, various combinations of threshold values were tested to find the best one. From the datasets (Kvasir [17] and Nerthus [16]) of more than 10,000 images (16 classes), the F1 score of 0.907 and MCC (Matthew correlation coefficient) score of 0.952 were reported.

In [134], five methods in which each method is a different combination of existing classifier(s) were proposed. In Method 1, the supervised learning classifier from Weka software [135] to build a linear logistic regression model was combined with LogitBoost [136]. In Method 2, the Logistic Model Tree classifier from Weka software was used. Method 3 used only ResNet-152 [28]. In Method 4, ResNet-152 was combined with DenseNet-161 [115] using simple averaging of the final class probabilities. In Method 5, multi-layer perceptron (MLP) was used to combine the outputs from ResNet-152 and DenseNet-161 instead of the simple averaging because simple averaging does not produce an accurate classification when the two models provide different outcomes. ResNet-152 and DenseNet-161 were trained separately, and the MLP was trained using their outputs. These five methods were evaluated on the 2018 Medico dataset [137], CVC-356-plus (a modified version of CVC-356 [138]), CVC-612-plus (a modified version of CVC-612 [138]), and CVC-12k [139]. MCC scores of 0.63 to 0.94 were reported as results.

A two-stream model for endoscopic image analysis, which fuses two streams of deep feature inputs by mapping their inherent relations through a relational network model, was proposed [140]. Extracted features from earlier layers and from later layers of the pre-trained CNN model were combined to facilitate the final prediction. Their accuracy, precision, recall, F1-score, and MCC were between 0.88 and 0.99 on two public datasets (Kvasir [17] and Nerthus [16]).

A two-stage knowledge distilled framework was proposed to detect polyp, Meckel’s diverticulum, ulcer, and bleeding in colonoscopy frames [141]. The accuracies between 83 and 94% on 3,799 colonoscopy images were reported. The accuracy for detection of Meckel’s diverticulum is better (around 13%) than the existing work, but the accuracy for detecting polyp, ulcer, and bleeding is very similar with the others. MobileNet from the Jetson-inference software package [142] was used [143] to classify sessile polyps, pedunculated polyps, lipoma, diverticulum, bleeding, vascularized mucosa, water jet, multi-tool head, forceps, and snare) in colonoscopy frames. Accuracy was not reported.

A semi-supervised learning approach using an unsupervised jigsaw learning task [144] in combination with supervised training (ResNet-18 [28]) was proposed in [145] to classify two classes: ‘neoplastic/precancerous’ and ‘non-neoplastic’ polyps. Using the histologic labels, adenomas and serrated adenomas were assigned to the neoplastic/precancerous class, while hyperplastic polyps were assigned to the non-neoplastic class. Several percentage improvement was reported in correctly classifying lesions when compared to a fully-supervised baseline.

### D. Detection of Biopsy and Therapeutic Treatment

Colonoscopy not only allows for detailed examination of the entire colon, but also removal of all premalignant lesions during the procedure. Most often diagnostic or therapeutic operations are performed during the withdrawal phase when instruments are inserted via a working channel within the shaft of the endoscope. A variety of instruments (e.g., forceps, snares, and cytology brushes, needles for sclerotherapy or mucosal injection, and aspiration catheters) can be used. Within a single procedure, the head and the cable of the instrument typically appear in the field of view (FoV) of the camera. Detection of operations is useful for obtaining more fine-grained quality metrics such as withdrawal time without time spent for treatment and quality of treatment.

Cao *et al.* investigated methods for detecting instrument images using hand-crafted features [146], [147]. The detected consecutive instrument frames are grouped to form an *operation shot* as a segment of visual data that corresponds to a diagnostic or therapeutic operation. The proposed methods were not fast enough to run in real-time [147]. Zhang *et al.* proposed a faster method for prediction of instrument frames and detects an *instrument scene* or *operation scene* defined as a video segment corresponding to a single purpose diagnostic or therapeutic action [148]. One scene may consist of one or more operation shots such as several biopsy shots taken in close proximity in the colon. This technique, although fast, also cannot be used in real-time. The aforementioned methods thus far use hand-engineered features of cable body and cannot detect when only instrument heads appear in the FoV since instrument heads have totally different appearances.

Zhang *et al.* introduced EndoCNN with four pairs of convolutional and pooling layers, followed by a fully connected layer and a softmax layer to classify four instrument classes and one non-instrument class [149]. The authors also proposed a similarity-based data augmentation method that recommends selected unlabeled images for manual labeling to add to the seed training dataset. On the test dataset of 36,210 images, the average F1 score is 0.95 when using the similarity-based data augmentation to expand a small seed training dataset to 52,000 images. The model can run in real-time and detect instruments when only the head portions of instruments are visible as well.

## IV. Clinical Trials With Real-Time AI-Assisted Colonoscopy

Although AI for colonoscopy has received much research attention over the years, there have been relatively few systems tested in clinical trials. There are nine reports of clinical trials of real-time AI-assisted colonoscopy, seven single-center [39], [150]–[155] and two three-center clinical trials [156], [157]. Four trials [39], [151], [154], [156] provided feedback on quality of colonoscopy. The systems in the remaining four trials provided feedback solely for polyps. All these trials show that AI-assisted systems improve colonoscopy outcome either by increasing quality or detecting more polyps. The first clinical trial reported in 2012 used EMIS software that detected the start and end of each procedure automatically in real-time. It measured multiple intra-procedure quality metrics: clear withdrawal time without blurry frames, amount of stool seen on images during insertion and withdrawal, BBPS scores, and the spiral score. The provided feedback consisted only of the aforementioned “spiral score.” In [150], a sound alert was made when computer automated detection (CADe) [85] detected a polyp. The detected polyp bounding box was shown on a second monitor. In a later trial using the same CADe system, the same type of feedback was shown directly on the diagnostic monitor [153]. CADe design is based on SegNet [86], a deep learning method for image segmentation. In the trial reported by [157], GI-Genius provided a green prompt surrounding the detected polyp region on the diagnostic screen. The trial by Su *et al.* [151] used audio prompts when continuous blurry frames were detected. The detected polyp location was displayed on a second monitor. Su *et al.* utilized five neural-network models, four of which used features extracted from existing pre-trained models as input to shallow fully-connected neural networks [151]. They predicted cecum images to identify the beginning of the withdrawal phase of a colonoscopy, removal of the endoscope from the patient, BBPS scores, and withdrawal stability through prediction of blurry frames and similarities between frames, respectively. For polyp detection, the DL model based on YOLOv2 [69] was used.

Gong *et al.* [154] used EndoAngel to monitor withdrawal speed and colonoscopy withdrawal time using three CNN models. A warning was presented when endoscope slipping was detected (continuous blurry frames). Ten frames prior to the beginning of the slipping were displayed at the bottom of the screen until pictures similar to the ten frames were detected. The authors did not elaborate whether the frames were shown on the same diagnostic screen or another monitor. A nurse pushed a button to indicate the start of the withdrawal time if ten consecutive frames showing cecum images were not detected. The trial by Maeda *et al.* [155] required the use of endo-cytoscope and Narrow Band Imaging to study the effectiveness of their AI-system on predicting ulcerative colitis activity.

Current feedback commonly used in clinical trials shows bounding boxes surrounding detected polyps, but not the detailed polyp contours. Under ideal circumstances, polyps are removed with a margin of normal tissue surrounding the polyp; therefore polyp detection is more important than polyp segmentation. Yet, segmentation may be critical to assess completeness of resection.

## V. Future of AI for Colonoscopy

Leading domain experts are optimistic about the prospective of using AI systems in daily practice for real-time assistance during colonoscopy [7]–[11]. However, they have reservations regarding three issues: robustness, transparency, and integration with clinical workflow. We will examine the two former issues in more detail but limit the discussion of the latter given the breadth of the topic. Lastly, we will briefly discuss the potential of AI as a driver of autonomic or robotic instruments.

As DL systems are prevalent technologies for AI for colonoscopy, availability of large ground truth datasets under optimal and sub-optimal conditions is critical to advance the performance of AI assisted systems. And even with availability of optimal ground truth datasets we must define the boundaries of valid use and realize when AI-based results may have limited value. Models based on private datasets will need to disclose the patterns the models recognize and the prevalence of these patterns in the datasets. This will help to understand the limitations of the models trained on such private datasets.

### A. Robustness

The effectiveness of specific CNNs is highly dependent on the training dataset [158]. Obtaining a sufficiently large and representative training dataset of population data during routine colonoscopy screening is difficult due to variations in colon anatomy, the quality of colon preparation, the navigation and inspection techniques of endoscopists, presence of unknown type and degree of disease, and endoscopists’ intervention techniques and skill sets. Moreover, manual labeling of training data by domain experts is very expensive. Significant class imbalance is often found (i.e., images of the class of interest occur infrequently). For instance, the class imbalance ratio of images of non-instrument versus instrument class is about 44:1 for the instrument image classification problem [159]. What makes classification even more challenging is the fact that the common class may contain a great variety of image patterns. Significant class imbalance if not properly handled results in incorrect prediction of rare class images. Hence, creating a representative training dataset is critical and, unfortunately, often time-consuming.

Common approaches that have been applied to improve robustness of DL for colonoscopy are as follows.

- Synthetic Data Augmentation (SDA): SDA is the most commonly used method to improve image classification. SDA synthetically generates multiple variations of a training image. The most common SDA approach for colonoscopy applies user-specified image transformation methods such as rotation, zooming in/out, and cropping and translation [160]. SinGAN-Seg [161] is a generative adversarial network-based method recently proposed to generate synthetic images for polyp segmentation.
- Active Learning (AL): Given a small initial training dataset, AL methods minimize manual labeling efforts by using a query strategy to select necessary sample images (typically from an unlabeled dataset) for the domain experts to classify. A new classifier is constructed from the enlarged training dataset. This process is repeated until a stopping criterion is satisfied. Several query strategies were explored (e.g., selecting samples at the border separating different classes and selecting outlier samples). Zhou *et al.* [162] and Zhang *et al.* [159] proposed AL methods that were tested on colonoscopy datasets.
- A few-shot learning method trained on a large number of normal images and fewer abnormal images (less than 100 frames) was applied on polyp image detection [163].

More advanced SDA methods (e.g., linear and non-linear mixing of randomly cropped, labeled images and feature space augmentation) have been shown to improve classification performance for generic images [164]. Nevertheless, SDA methods are inherently limited by patterns in the training dataset before augmentation. Given a large unlabeled dataset from routine colonoscopy screening, effective AL methods are more likely to add diverse patterns seen in practice. Recent AL methods utilizing variational auto-encoder were proposed [165], [166]. Other approaches are 1) semi-supervised learning [167], [168], 2) zero and few-shot learning [169]–[171], and 3) domain-specific or out-of-domain transfer learning via supervised learning on annotated medical data [129], [172], [173].

### B. Transparency of DL Models

DL methods automatically extract important image features from the training data and build the function for prediction using the extracted features. It is important to be able to determine for a given image whether the correct pixels/regions or features are used to predict the class assigned to the image. **Local interpretation** explains the prediction decision for a given image. **Global interpretation** explains an entire DL model, giving all patterns the model can recognize or patterns detected by individual or groups of neurons in various layers of the model. Global interpretation reveals the overall capability and limitations of a model. Ideally, local and global explanations are readily available, convincing and easy to understand. Limitations of a DL model may include causes such as the choice of model, training data that do not represent the target population, biases in the training data, and labeling errors. Interpretation tools may show some of the limitations of a DL model, and therefore may be useful for clinical decision makers as they provide some insights in the DL “black box” while reviewing different AI systems for possible implementation in clinical practice. For endoscopists these tools may help understand the DL classification process by showing evidence in support of or against DL-based recommendations.

#### 1) Local Interpretation:

We divide local interpretation methods into two sub-categories: pixel-based interpretation and concept-based interpretation.

**Pixel-based interpretation** methods assign relevance scores to individual pixels to reflect how well they support the predicted class and output the heatmap of the relevance scores. The heatmap does not explicitly convey relationships between highly relevant pixels and corresponding semantic concepts in the images of the predicted class because we do not know what features in the training data cause the final classification. Pixel-based interpretation methods mostly work on image classification problems except for a single work [174] that applied this method to polyp segmentation. Since image segmentation decides which pixel belongs to which region, the interpretation should inform the reasons for selection or rejection of the pixel as part of region of interest, such as a polyp. Three main approaches for computing the relevance scores for image classification are as follows.

- **Relevance score backpropagation** methods obtain the output score of the predicted class and redistribute the score via backpropagation to the input layer. Examples include Layer-wise Relevance Propagation (LRP) [175] and Class Activation Map (CAM) [132].
- **Gradient based** methods calculate relevance scores of individual pixels as the absolute values of the gradients of the predicted class score with respect to a given input image [176]. Gradient-by-input methods calculate the relevance score of each pixel by multiplying the gradients by the output of a particular convolution layer. If needed, up-sampling the generated heatmap to the size of the input image is applied. Grad-CAM [31], Grad-CAM++ [177], DeepLIFT [178], Deep Taylor [179], and Integrated Gradient [180] are examples of this approach. These methods have received much research attention in recent years as they are applicable to any CNN architecture and allow fast interpretation calculation via a single backpropagation. In general, as the gradient based methods identify the most discriminative pixels in an input image, the interpretation output may cover only part of the discriminative object in the image, making it difficult to understand the basis for the classification.
- **Attention-based** methods learn the weights of an attention map to get the classifier to focus more on the relevant parts of the input for classification. The learned attention map is then used to create a heatmap of the relevant pixels in the input image [181]. Training the attention map adds additional computational cost, but the interpretation of a test image is fast. However, the effectiveness of the attention-based interpretation has not yet been proven [182], [183].

**Fig. 4.**
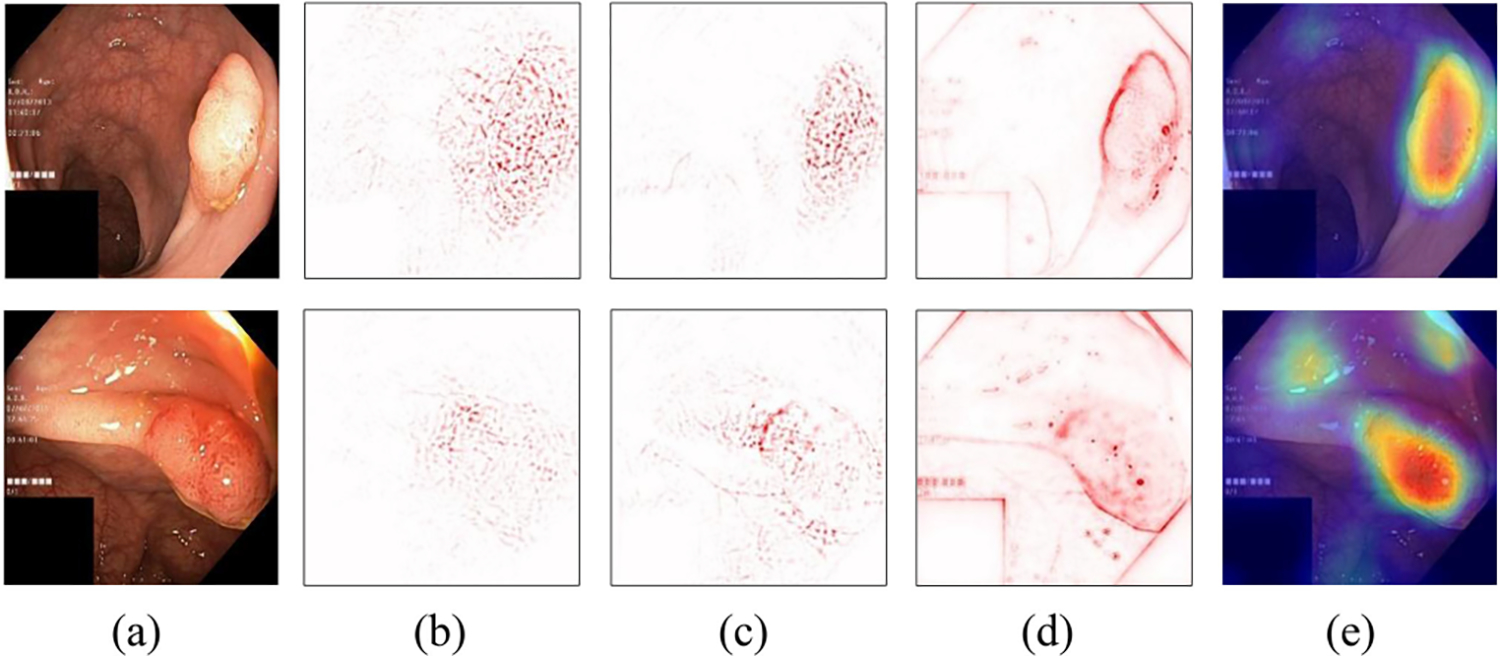
Examples of pixel-based interpretation on polyp images of the Kvasir V2 public dataset [17]: (a) input image, (b) gradient, (c) LRP, (d) Deep Taylor, and (e) Grad-CAM.

Fig. 4(b)–(e) show pixel-based interpretation examples by various methods for polyp image classification of Fig. 4(a). The redder the pixels are, the likelier these pixels are used by the classifier to predict the image as a polyp image. However, the interpretations do not provide insight about which edge, color, or shape patterns determine image classification. We also do not know how well such patterns are represented in the training data; are they representing rare or commonly seen polyps? That type of information is useful to improve confidence in classification results.

**Concept-based interpretation** methods highlight regions that represent similar concept(s) learned from the training data for the predicted class. This approach provides some knowledge about the interpretation and the relevant training data. For instance, Li *et al.* proposed to learn image-level prototypes (representation of concepts in the training data) for a DL classifier by minimizing classification loss, image reconstruction loss, and loss reflecting the distance between the learned image prototypes and training images [184].

##### • Self-interpretable classification models:

These models learn to automatically extract prototypes or generalized representations of a class and use them for both classification and interpretation [185]–[187]. The prototype based self-interpretable deep classification model has a tendency to offer slightly lower classification accuracy compared to a non-prototype approach as found by the authors of [187] and [188]. Improving of accuracy can be achieved via other means such as transfer learning.

##### • Contrastive explanation:

The methods in this category present images most similar to the input image but of a different class. Given image regions and corresponding text explanation for each training image, neural networks were trained to select the most suitable contrastive explanation [189], [190].

##### • Hierarchical interpretation:

**Fig. 5.**
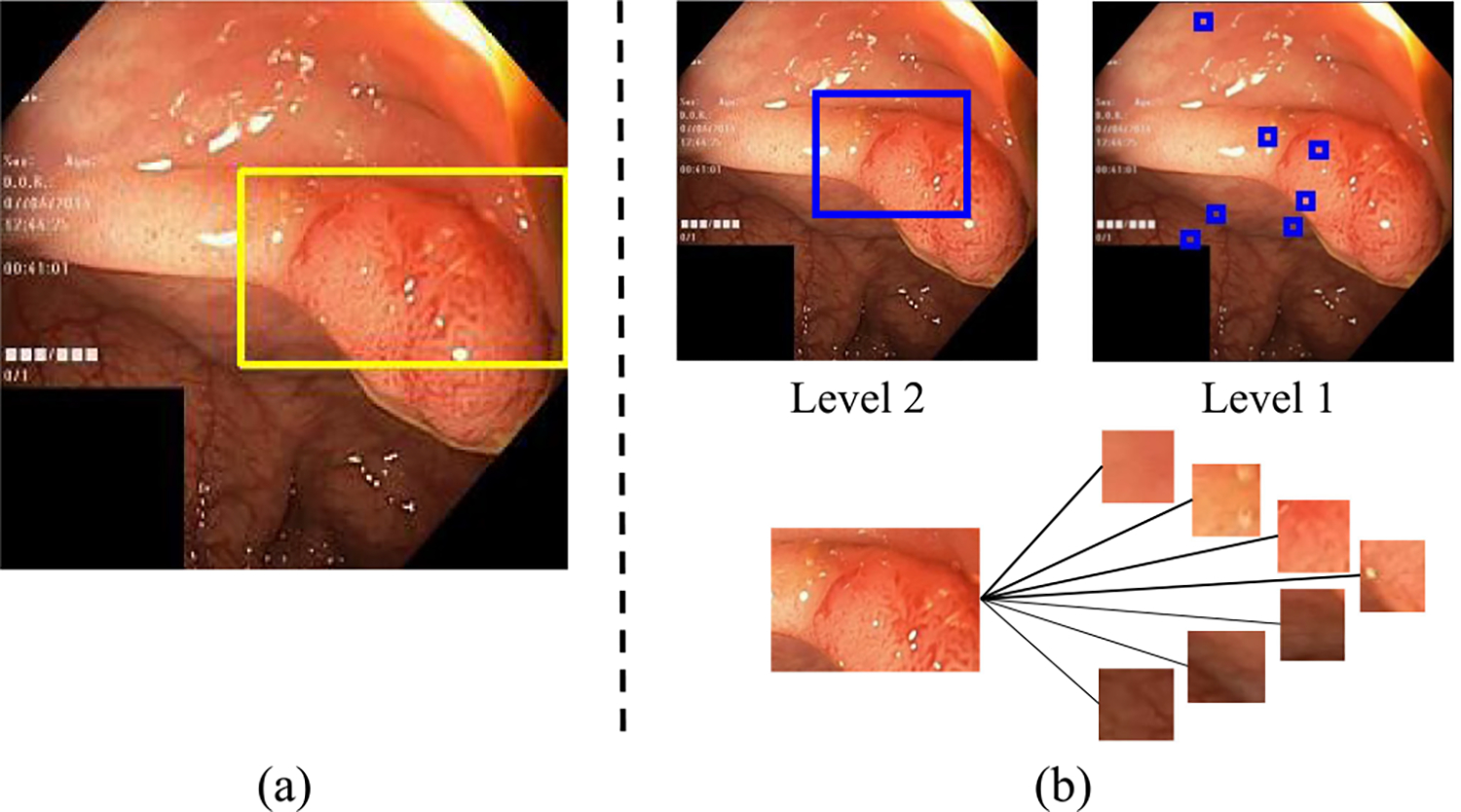
Examples of (a) ProtoPNet [187] and (b) two-level hierarchical concept-based interpretation [188] on polyp images of the Kvasir V2 public dataset [17]. The blue and yellow boxes indicate the image object that is used for prediction. Level 2 shows the object level concepts (e.g., the polyp object). Level 1 shows the low-level concepts (different shades of red colors and texture) that make up the polyp object. Thicker connecting lines indicate stronger influence of the lower-level to higher-level concepts.

Wang *et al.* [191] used a manually labeled dataset [192] of color, texture, objects, scenes for generic objects to build a hierarchy of concepts for image-level and class-level interpretation. Their method requires that each concept has a set of binary segmentation mask images and the concept label as ground truth. The method cannot detect other concepts beyond the ones manually labeled. Khaleel *et al.* developed a method that automatically learns concepts at different semantic levels (e.g., color, texture, and object) from the training dataset [188] and produces a hierarchy of the concepts found in a test image as interpretation. Fig. 5 shows concept-based interpretation examples.

#### 2) Global Interpretation:

The methods in this category attempt to reveal what image properties the neural network neurons or layers detect or what patterns the model recognizes. Recent global interpretation methods are described in survey [193]. Zeiler and Fergus proposed a visualization method that shows patterns detected at intermediate layers by applying deconvolution and un-pooling operations [194]. Their method does not reveal relationships among the patterns across layers beyond spatial locations. Ghorbani *et al.* proposed to construct high-level concepts that are meaningful to humans, and coherent and important for classification [195]. Bau *et al.* proposed to dissect a CNN network by identifying which neurons in the CNN detects which concept using the intersection-over-union score between the predicted and ground truth mask [192].

To the best of our knowledge, there are no large studies that objectively evaluate any of these interpretation methods with leading domain experts in gastroenterology. We believe that global interpretation that provides patterns recognized by a model will be useful for adoption of an AI system. Local interpretation is useful for a retrospective review of performance of an AI system.

### C. Integration Within Clinical Workflow

There are several potential benefits of integrating AI into the clinical workflow. First, presenting AI-generated information obtained in real-time during colonoscopy to the endoscopist is critical to improve outcome of the patient who is undergoing the procedure. Any feedback at that time potentially can change endoscopist behavior. The current focus in colonoscopy is on detection and segmentation of possible polyps, and the classification of detected polyps in likely benign or pre-malignant class. However, polyp detection, segmentation and classification can only occur for lesions within the field of view. AI can also provide information about areas of the colon not well or not at all seen [37], [47]. This information, when presented in a timely fashion, can stimulate the endoscopist to improve image clarity, remove remaining fecal matter or reposition the endoscope tip to allow visualization behind haustrae or sharp angulations. At present we do not know what information should be presented to the endoscopist, in what format, where on the monitor, and for how long.

Second, the information obtained can be used to pre-fill an endoscopy report; current endowriters require extensive clicking of entry fields to provide detailed information about preparation, findings, interventions and complications. Future methods will be able to determine all of this, select and mark appropriate image or video documentation, and document all within structured and at the same time in a easily readable format for humans. This will result in more complete procedure documentation and allow more time for actual patient-physician contact at potentially lesser cost. AI can also be used to objectively score inflammatory bowel diseases to allow comparison of patients seen by different endoscopists among centers anywhere in the world; this allows a single universal classification which may facilitate treatment optimization for patients with inflammatory bowel diseases and accelerate clinical trials of new drugs in these diseases [109], [196].

Third, AI-based information provides objective information about the quality of individual endoscopist or an endoscopy group, the average colon preparation of the patient population, the amount of time patients are within colonoscopy, disease trends of patients seen, number and type of specific instruments used, etc. Thus ample information will become available to manage endoscopic skill sets among the endoscopy team members, optimize schedules, manage the practice, maintain adequate supplies and predict practice trends.

### D. Autonomic and Robotic Instruments

Knowledge of location of the endoscope tip and the location and nature of any lesions allows steering of endoscope and instruments. We foresee a gradual introduction of DL-based automation, initially under direct human supervision. Eventually standalone instruments completely driven by autonomous software may result in colonoscopy robots [197]. For instance, current manipulation of the endoscope tip is manually via dials in order to steer the tip of the endoscope in the direction of the upstream lumen; there is no reason to believe that DL cannot do this as well if not better than human operators. Patient movement, breathing and pulsating heart or vessels may move the endoscope tip away from a polyp that needs to be removed; DL-based software may automatically correct for these movements facilitating complete polyp removal. Current video capsule endoscopy does not allow steering of the capsule, obtaining samples or remove lesions; all of this in theory can be addressed, and DL is expected to play a major role in this [3]. With miniaturization and better battery technology any hardware can be located inside the body whereas the software driving a robotic capsule able to change position or remove lesions is residing outside the patient. Indeed, it is likely that predominantly hybrid robots will be applied in the colon where the tools are inside and the operating system outside the patient, either connected via a wire, also allowing power transmission, such as via the anus, or a wireless solution, requiring a battery-operated robot [198].

## VI. Conclusion

We present a summary of research over the past two decades and the progress made towards real-time AI-assisted colonoscopy. Recent clinical trials have shown that feedback during live procedures improves quality of patient care by detecting more polyps. More work is to be done as described in the future research directions. Data privacy complicates matters as sharing of detailed medical image data is not allowed. Finally, having all the tools and implementing them in clinical practice does not mean that the problem at hand is solved. Perfect AI-scores for cleaning and circumferential inspection of the colon are not the same as having carefully inspected all mucosa. All it means is that the endoscopist has met the expectations of the AI-based classifiers. What eventually is needed are trials that show that AI-based techniques implemented during colonoscopy lower the incidence, morbidity and mortality of CRC [20]. Those have been and will continue to be the ultimate indicators of successful CRC prevention; therefore AI assisted systems need to show that their implementation lowers these CRC benchmarks.
